# Healthcare-associated infections in intensive care units in Taiwan, South Korea, and Japan: recent trends based on national surveillance reports

**DOI:** 10.1186/s13756-018-0422-1

**Published:** 2018-11-07

**Authors:** Cho-Han Chiang, Sung-Ching Pan, Tyan-Shin Yang, Keisuke Matsuda, Hong Bin Kim, Young Hwa Choi, Satoshi Hori, Jann-Tay Wang, Wang-Huei Sheng, Yee-Chun Chen, Feng-Yee Chang, Shan-Chwen Chang

**Affiliations:** 1 College of Medicine, National Taiwan University, Taipei, Taiwan; 2 Department of Internal Medicine, National Taiwan University Hospital, Taipei, Taiwan; 3 Faculty of Medicine, Osaka University, Osaka, Japan; 4 Department of Internal Medicine, Seoul National University College of Medicine, Seoul, Republic of Korea; 5 Division of Infectious Diseases, Seoul National University Bundang Hospital, Seongnam, Republic of Korea; 6 Department of Infectious Diseases, Ajou University School of Medicine, Suwon, Republic of Korea; 7 Department of Infection Control Science, Juntendo University Faculty of Medicine, Tokyo, Japan; 8 Center for Infection Control, National Taiwan University Hospital, Taipei, Taiwan; 9 National Institute of Infectious Diseases and Vaccinology, National Health Research Institutes, Miaoli County, Taiwan; 10 Division of Infectious Diseases and Tropical Medicine, Department of Internal Medicine, Tri-Service General Hospital, National Defense Medical Center, Taipei, Taiwan

**Keywords:** Healthcare-associated infections, National surveillance, Antimicrobial resistance, National policy, Infection prevention and control program

## Abstract

**Background:**

Sustainable systematic interventions are important for infection prevention and control (IPC). Data from surveillance of healthcare-associated infections (HAI) provides feedback for implementation of IPC programs. To address the paucity of such data in Asia, we searched for national HAI surveillance and IPC programs in this region.

**Methods:**

Data were analysed from open access national surveillance reports of three Asian countries: Taiwan, South Korea and Japan from 2008 to 2015. National IPC programs were identified.

**Results:**

There were differences among the countries in surveillance protocols, hospital coverage rates, and national IPC policies and programs. Nevertheless, there was a 53.0% reduction in overall HAI over the 8-year period. This consisted of a decrease from 9.34 to 5.03 infections per 1000 patient-days in Taiwan, from 7.56 to 2.76 in Korea, and from 4.41 to 2.74 in Japan (Poisson regression, all *p* < 0.05). Across the three countries, *Escherichia coli* and *Candida albicans* were the major pathogens for urinary tract infection. *Staphylococcus aureus, Acinetobacter baumannii* and *Enterococcus faecium* were common bloodstream pathogens. For pneumonia, *S. aureus*, *A. baumannii*, *Pseudomonas aeruginosa*, and *Klebsiella pneumoniae* were the predominant pathogens, with considerable country differences. There was a 64.6% decrease in the number of isolates of methicillin-resistant *S. aureus*, 38.4% decrease in carbapenem-resistant *P. aeruginosa* and 49.2% decrease in carbapenem-resistant *A. baumannii* (CRAB) in Taiwan (all *p* < 0.05), and similarly in Korea with the exception of CRAB (30.5 and 50.4% reduction, respectively, both *p* < 0.05**)**.

**Conclusion:**

We found a significant decrease in HAI across the three countries in association with sequential multifaceted interventions such as hand hygiene, care bundles, and antimicrobial stewardships. Further regional collaboration could be forged to develop joint strategies to prevent HAI.

**Electronic supplementary material:**

The online version of this article (10.1186/s13756-018-0422-1) contains supplementary material, which is available to authorized users.

## Background

The European Healthcare-associated Infections Surveillance Network (HAI-net) is one of the most coordinated and comprehensive surveillance systems that monitors healthcare-associated infections (HAI). By centralizing data on antimicrobial use, HAI incidence, and HAI point prevalence, HAI-net builds a regional landscape that allows inter-country comparison and provides feedback for implementation of regional infection prevention and control (IPC) guidelines [1].

With its high burden of HAI, Asia stands to benefit by learning from such a surveillance network. A recent meta-analysis reported a pooled HAI incidence density of 20 cases per 1000 intensive care unit-days in Southeast Asia [2]; studies in India and China found pooled ventilator-associated pneumonia of 9.4 and 20.8 cases per 1000 ventilator-days, respectively [3, 4]. Establishing surveillance in Asian countries, either as national or regional collaborations, might help relevant stakeholders to identify systemic gaps and establish improvements in IPC.

The current understanding of HAI surveillance in Asia remains limited despite the relatively large numbers of IPC conducted in Asia [2, 5]. Likewise, national scale data documenting the regional HAI epidemiology in Asia is scarce [2]. To better understand the current state of HAI surveillance and IPC programs in Asia, we searched for data on existing national HAI surveillance programs. Three Asian countries: Taiwan [6, 7], South Korea [8–10], and Japan [11] were found to conduct nationwide HAI surveillance systems. The present study is based on data derived from open access reports from the surveillance systems of these countries. They include temporal trends of HAI in intensive care units (ICUs), the major causative pathogens and antimicrobial resistance (AMR). Nationally implemented IPC policies were also reviewed to gain insights on important interventions instituted in these three countries.

## Methods

### Study design and source of data

We performed a Google and PubMed search to determine the existing national HAI surveillance systems in Asian countries using the following terms “national nosocomial infection surveillance” or “national healthcare-associated infection surveillance” in combination with specific country names. The inclusion criteria were: English language, open access data or PubMed publications, annual data containing either point prevalence or yearly surveillance for 5 or more years. Data from the national HAI surveillance systems were retrospectively retrieved and analysed.

### National surveillance systems of Taiwan, South Korea, and Japan

Three national HAI surveillance systems met the study criteria. These were the Taiwan Nosocomial Infection Surveillance (TNIS), Korean National Healthcare-associated Infection Surveillance (KONIS), and Japan Nosocomial Infection Surveillance (JANIS). Each system prospectively collects data on the incidence, causative pathogens, and antimicrobial resistance of HAI in ICUs. HAI data are stratified by infection site: urinary tract infection (UTI), bloodstream infection (BSI), hospital-acquired pneumonia (HAP); by device-use: catheter-associated urinary tract infection (CAUTI), central line-associated bloodstream infection (CLABSI), and ventilator-associated pneumonia (VAP); and by type of hospital (in Taiwan and South Korea). These HAI cases and categories are in accord with the definitions of the US National Healthcare Safety Network (NHSN) system with minor modifications to account for differences in clinical or laboratory practice and national policies.

### Data collection

Demographic data for each country were retrieved from the World Bank and their respective national authorities. Hospital and ICU composition of each surveillance system were recorded from their official web portals. Annual data of overall HAI, device-associated HAI, causative pathogens, and rates of AMR of important bacteria were also retrieved from the three surveillance systems. We selected the study period as 2008 to 2015 because data for this period were accessible across all three systems. National-scale IPC policies and programs were obtained by online search or in consultation with experts from the three countries.

### Data analysis

Incidence densities of overall HAI were determined as pooled means of UTI, BSI, and HAP rates, and calculated as overall HAI episodes per 1000 patient-days. Analysis of device-associated HAI included CAUTI, CLABSI, and VAP. For Taiwan and Korea, incidence densities of device-associated HAI were calculated as device-associated infection episodes per 1000 device-days. For Japan, device-associated HAI were analysed by device-associated infection episodes per 1000 patient-days which made Japanese data incompatible with data from other countries. Causative pathogens were classified at the species level. AMR proportions of selected pathogens were calculated as number of antimicrobial-resistant isolates divided by the total number of isolates of the same species.

### Statistical analysis

A Poisson regression model was used to assess the temporal trends of HAI incidence. Linear regression was used to analyse the trends in AMR isolates, using the STATA statistical program (version 14.0 Texas, USA). A *P* value < 0.05 was considered statistically significant.

## Results

### Characteristics of Taiwan, South Korea and Japan’s National Surveillance Systems

The characteristics of the national HAI surveillance systems of Taiwan, South Korea, and Japan are summarised in Table 1 [6, 8, 11]. The type, size and proportion of hospitals enrolled in the national surveillance varied among the countries. Taiwan included medical centres and regional hospitals classified according to hospital accreditation. Most of them had hospital beds of 300 beds or more. Korea and Japan included hospitals with more than 300 and 200 beds, respectively. The hospital coverage and participation rates were 21.2 and 100.0% in Taiwan, 18.0 and 38.6% in Korea, and 1.9 and 6.8% in Japan, respectively. A total of 472, 169 and 163 ICUs were enrolled in Taiwan, Korea and Japan, respectively. The number of participating hospitals and ICUs in all three countries during the study period has expanded (Additional file 1: Table S1) [6, 8, 11]. Categorization of HAI was different in JANIS, which presented only UTI, CLABSI and VAP. Infection incidence was also calculated differently as episodes per 1000 patient-days.

**Table 1:** Demographics and national surveillance systems of Taiwan, South Korea and Japan

Parameter	Taiwan	South Korea	Japan
Country background			
Population^a^	23,433,753^b^	50,746,659^c^	127,276,000^d^
Income bracket^e^	High income	High income	High income
GDP, US dollars	571,736 million^f^	1,530,750.92 million^g^	4,872,136.95 million^g^
Share of GDP on national health expenditure	6.3%^f^	7.6%^h^	10.7%^h^
Number of hospitals^a^	486^i^	534^j^	7426^k^
Surveillance system	Taiwan Nosocomial Infection Surveillance (TNIS)	Korean National Healthcare-associated Infection Surveillance System (KONIS)	Japan Nosocomial Infection Surveillance (JANIS)
Year established	2001	2006	2000
Authority	Centers for Disease Control, Ministry of Health and Welfare, Taiwan	Korea Centers for Disease Control and Prevention	Ministry of Health, Labor and Welfare, Japan
ICU Surveillance^a^			
Number of hospitals enrolled	103	96	143
Number of ICUs enrolled	472	169	163
Types of hospitals enrolled (total number in the country)	Medical Centers and Regional hospitals ^l^ (*n* = 103)	Bed size > 900, 700–899, 300–699 (*n* = 249)^n^	Bed size > 200 (*n* = 2100)
Hospital coverage rate	21.2% (103/486)	18.0% (96/534)	1.9% (143/7426)
Hospital participation rate^m^	100.0% (103/103)	38.6% (96/249)	6.8% (143/2100)
Mandated standard ratio of infection control personnel	1 dedicated full-time certificated IC nurse per 300 beds (basic) or per 250 beds (optimal) 1 FTE qualified IC doctor per 500 beds (basic) or per 300 beds (optimal)^o^ For hospitals > 500 beds: 1 FTE IC medical technician (basic) or 1 dedicated full-time certificated IC medical technician (optimal); 1 FTE IC medical technician for hospitals with 300–499 beds (optimal)	1 dedicated full-time IC nurse per 200 beds (basic) or per 150 beds (optimal)^n^ 1 qualified IC doctor per 300 beds	1 dedicated full-time certificated IC nurse (at > 0.8 FTE)^p^ 1 part-time IC doctor (at > 0.5 FTE) 1 part-time IC medical technician and 1 part-time pharmacist (at > 0.5 FTE) Additional manpower for antimicrobial stewardship^p^
Healthcare-associated infection data provided			
Site-specific HAIs	UTI, BSI, HAP: episode per 1000 patient-day	UTI, BSI, HAP: episode per 1000 patient-day	UTI: episode per 1000 patient-day
Device-associated HAIs	CAUTI, CLABSI, VAP: episode per 1000 device-day	CAUTI, CLABSI, VAP: episode per 1000 device-day	CLABSI, VAP: episode per 1000 patient-day
Causative pathogens	Top 10 of the most common pathogens	99% of all the causative pathogens	Top 5 of the most common pathogens^q^
Antimicrobial-resistant pathogens	MRSA, VRE, CRAB, CRPA, CRE, CREC, CRKP	MRSA, VRE, IRAB, IRPA, CefR-KP, CipR-KP, CefR-EC, CipR-EC	MRSA

*Abbreviations: BSI* bloodstream infections, *CAUTI* catheter-associated urinary tract infection, *CefR-EC* cefotaxime-resistant *Escherichia coli*, *CefR-KP* cefotaxime-resistant *Klebsiella pneumoniae*, *CipR-EC* ciprofloxacin-resistant *E. coli, CipR-KP* ciprofloxacin-resistant *K. pneumoniae*, *CLABSI* central line-associated bloodstream infections, *CRAB* carbapanem (imipenem or meropenem)-resistant *Acinetobacter baumannii*, *CRE* carbapanem (imipenem, meropenem, or ertapenem)-resistant Enterobacteriaceae, *CREC* carbapanem (imipenem, meropenem, or ertapenem)-resistant *E. coli*, *CRKP* carbapanem (imipenem, meropenem, or ertapenem)-resistant *K. pneumoniae*, *CRPA* carbapanem (imipenem or meropenem)-resistant *Pseudomonas aeruginosa*, *FTE* full-time equivalent, *GDP* gross domestic product, *HAI* Healthcare-associated infections, *HAP* hospital-acquired pneumonia, *IC* infection control, *IRAB* imipenem-resistant *A. baumannii*, *IRPA* imipenem-resistant *P. aerugonisa*, *MRSA* methicillin-resistant *Staphylococcus aureus*, *MSSA* methicillin-susceptible *S. aureus*, *UTI* urinary tract infections, *VAP* ventilator-associated pneumonia, *VRE* vancomycin-resistant enterococci (*Enterococcus faecalis* or *E. faecium*)

^a^2014 data

^b^Data retrieved from http://www1.stat.gov.tw/ct.asp?xItem=15408&CtNode=4692&mp=3. Assessed 14 April 2018.

^c^Data retrieved from https://data.worldbank.org/country/korea-rep. Assessed 14 April 2018

^d^Data retrieved from https://data.worldbank.org/country/japan?view=chart. Assessed 14 April 2018

^e^Data retrieved from World Bank Country and Lending Groups at https://datahelpdesk.worldbank.org/knowledgebase/articles/906519-world-bank-country-and-lending-groups. Accessed 10 September 2018. For the current 2019 fiscal year, high-income economies are those with a gross national income per capita, calculated using the World Bank Atlas method of $12,056 or more

^f^2016 data. Raw data NT dollars 17,152,093 million, converted to US dollars by ratio 30:1. Retrieved from https://www.mohw.gov.tw/lp-3781-2.html. Accessed 10 September 2018

^g^2017 data based on World Bank national accounts data, and Organization for Economic Co-operation and Development (OECD) National Accounts data. Retrieved from https://data.worldbank.org/indicator/NY.GDP.MKTP.CD. Accessed 10 September 2018

^h^2017 data based on Organization for Economic Co-operation and Development (OECD) estimated data for Japan and provisional data for Korea. Retrieved from https://stats.oecd.org/Index.aspx?DataSetCode=SHA. Accessed 10 September 2018

^i^Data retrieved from https://www.mohw.gov.tw/dl-40542-045687b7-aa43-458c-ab70-e8ff24c5b1b3.html. Accessed 10 September 2018

^j^Data retrieved from http://kosis.kr/eng/statisticsList/statisticsList_01List.jsp?vwcd=MT_ETITLE&parentId=D#SubCont. Accessed 10 September 2018

^k^Data retrieved from http://www.mhlw.go.jp/english/database/db-hh/2-2.html. Accessed 10 September 2018

^l^The data for Taiwan included medical centers and regional hospitals, which were classified according to hospital accreditation and covered only acute care hospitals

^m^The hospital coverage rate was calculated as the number of participating hospitals divided by the total number of hospitals in the same year in each country. The hospital participation rate was calculated as the number of participating hospitals divided by the total number of hospitals to be enrolled in each surveillance system

^n^In terms of surveillance, the requirement for participation in KONIS was 1 full-time infection control nurse over 200-bed size hospital. Regarding the mandatory personnel requirement, this regulation has been launched as a financial incentive program since 2016, as described in Additional file 2: Table S2

^o^Data available at https://www.cdc.gov.tw/professional/info.aspx?treeid=beac9c103df952c4&nowtreeid=bd387fa55fef03f0&tid=FED32554F2B55D11. Accessed September 10, 2018

^p^Infection prevention and control incentive through reimbursement policies was revised in 2010, 2012 and 2018, as described in Additional file 2: Table S2. Since 2012, each hospital is reimbursed 1000 JPY (about 10 USD) per patient per admission if it fulfills the Ministry of Health, Labor and Welfare requirements which mandated one dedicated full-time certificated ICN (at > 0.8 FTE), one part-time ICD (at > 0.5 FTE), one part-time IC pharmacist and one part-time medical technician/microbiologist (at > 0.5 FTE). Since 2018, reimbursement policies per admission included three parts. It provides 3900 JPY (about 39 USD) per admission for infection prevention and control incentive at a major hospital, or 1000 JPY for a small hospital. Additional 1000 JPY was reimbursed if this hospital participates a local IPC network incentive. Another 1000 JPY was reimbursed for AS incentive. For hospitals with AS incentive, it mandates the following manpower in addition to 2012 requirements: one part-time doctor mainly for AS (at > 0.5 FTE), one full-time ICP either a certificated ICN or IC pharmacist or medical technician

^q^MRSA and MSSA are listed as separate pathogens

### National infection control policies or programs and HAI trends across Taiwan, South Korea, and Japan

**Fig. 1:**
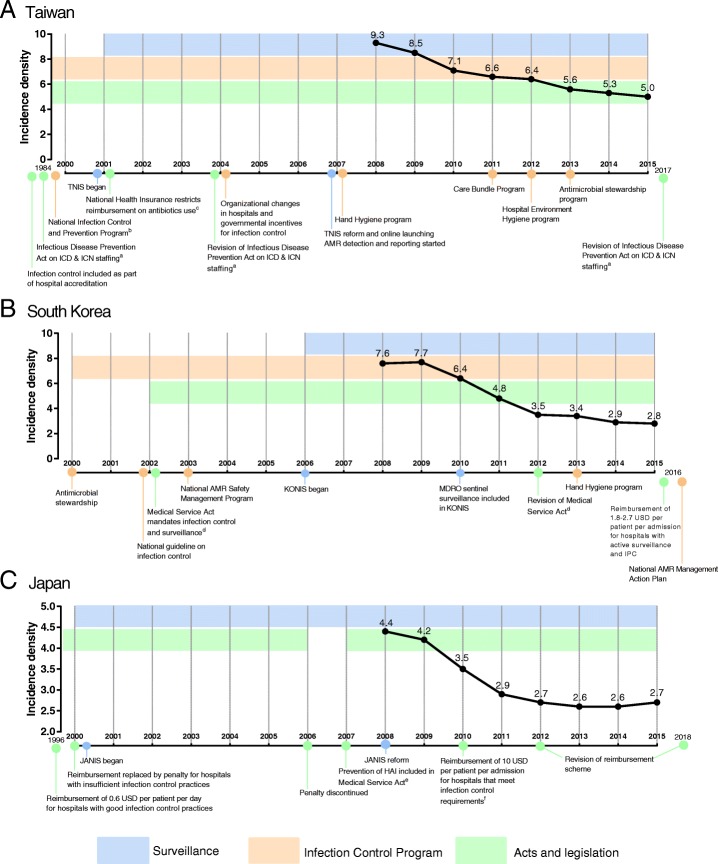
Incidence densities of healthcare-associated infections in intensive care units across Taiwan, South Korea, and Japan from 2008 to 2015. Abbreviations: AMR: antimicrobial-resistance; HAI: healthcare-associated infections; ICN: infection control nurse; ICP: infection control personnel; IPC: infection prevention and control; JANIS: Japan Nosocomial Infection Surveillance; ICD: infection control doctor; KONIS: Korean National Healthcare-associated Infection Surveillance; MDRO: multi-drug resistant organisms; TNIS: Taiwan Nosocomial Infection Surveillance. ^a^ In 1984, every teaching hospital in Taiwan was required to have one ICN per 300 hospital beds. In 2004, hospitals with more than 500 beds are required to have at least one ICD, and hospitals with more than 300 beds are required to have at least one ICN per 250 beds. In 2017, hospitals with more than 500 beds are encouraged to have one ICD for every 300 beds and one ICN for every 250 beds (Table 1). ^b^ Included training healthcare staff, establishing infection control committees, and formulating hospital policies. ^c^ Restricts use of antimicrobials in ambulatory patients with upper respiratory infections but without evidence of bacterial infection. ^d^ Act 29 specifies that IPC are the duties of hospitals with more than 300 beds. Act 47 mandates IPC as part of hospital accreditation. In 2012, hospitals with more than 200 beds are required to appoint an infection control committee and at least one full-time experienced staff (Table 1). ^e^ Japanese medical law obligated all health care institutions to implement operational safety measures against HAI, which includes IPC guidelines, IPC training, and disease reporting. ^f^ Hospitals should have an infection control team that consists of ICN, ICD, infection control pharmacist and infection control microbiology technologist. Hospitals should also have an IPC policy and antimicrobial stewardship program (Table 1). **a** Taiwan; **b** South Korea; **c** Japan

Numerous independent changes, aimed at improving surveillance and compliance, were made for national IPC policies, programs, or practices in each country (Fig. 1). Additional file 2: Table S2 summarised the details of national IPC programs in the past two decades by country. For example, during the study period hand hygiene, care bundles, hospital environment hygiene program and antimicrobial stewardship were the main interventions implemented in Taiwan. The hand hygiene program adapted the WHO multimodal strategies with particular emphasis on alcohol-based hand rub at the point of care. The care bundles program aimed to prevent CAUTI, CLABSI, and VAP. On the other hand, Korea and Japan enforced IPC practices by legislating and mandating IPC in hospitals. Incentives in terms of reimbursement 1.8–2.7 US dollars and 10 US dollars per admission were given to hospitals who met IPC standards in Korea and Japan, respectively. All three countries mandated assignment of infection control personnel. They also implemented formal and structured antimicrobial stewardship programs, which include surveillance of AMR pathogens and regulations of antimicrobial use.

### Overall HAI rates

All three countries experienced a significant reduction of approximately 50% in HAI rates by the end of the study period. The incidence density in Taiwan decreased by 46.2% from 9.3 to 5.0 infections per 1000 patient-days; in Korea HAI declined by 63.1% from 7.6 to 2.8, and in Japan by 38.6% from 4.4 to 2.7 (Poisson regression, all *p* < 0.05) (Fig. 1).

**Fig. 2:**
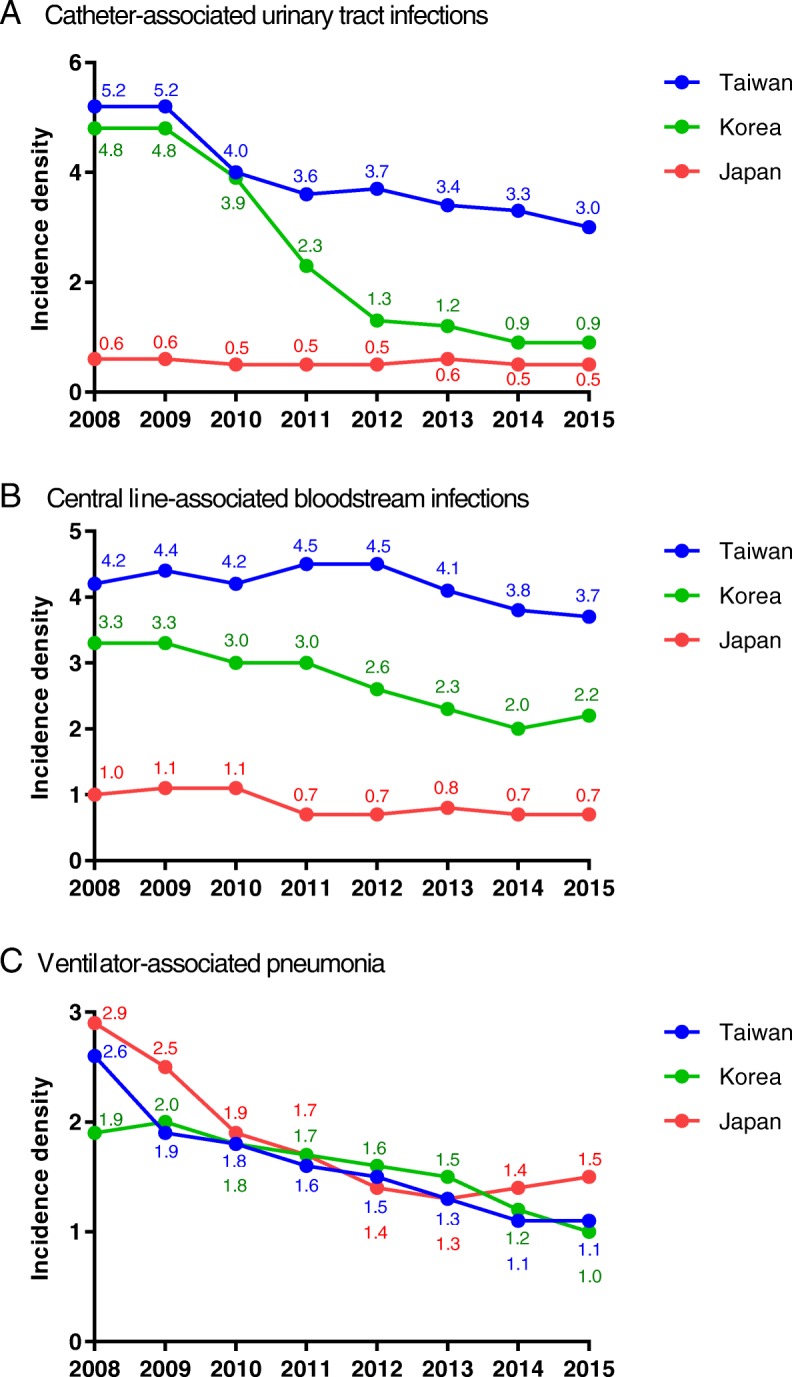
Annual trends of device-associated infections in intensive care units from 2008 to 2015. Data are presented as episodes per 1000 device-days (Taiwan, Korea) or episodes per 1000 patient-days (Japan; data comprised urinary tract infections, central line-associated bloodstream infections and ventilator-associated pneumonia). **a** Catheter-associated urinary tract infections; **b** Central line-associated bloodstream infections; **c** Ventilator-associated pneumonia

There was a significant reduction in device-associated HAI at all sites of infection in Taiwan and Korea (*p* < 0.05) (Fig. 2). Japan had low rates of CAUTI and CLABSI (presented as infections per 1000 patient-days) that persisted over the 8-year period. The most remarkable change was noted for CAUTI in Korea, with an 81.3% decrease from 4.8 to 0.9 infections per 1000 device-days (*P* < 0.05). All three countries experienced a similar trend in VAP during the study period with a 57.7% reduction from 2.6 to 1.1 infections per 1000 device-days in Taiwan.

### Causative pathogens

The distributions of the top five (or four for Japan) causative pathogens according to country and site of infection in 2015 are shown in Table 2. For Taiwan and Korea, UTI, BSI and HAP data were presented; for Japan, UTI, CLABSI and VAP data were presented. A more comprehensive list of pathogens is shown in Additional file 3: Table S3, Additional file 4: Table S4, and Additional file 5: Table S5.

**Table 2:** Common causative pathogens of healthcare-associated infections in intensive care units enrolled in the national surveillance systems of Taiwan, South Korea, and Japan in 2015

Rank	Organism	Proportion	Organism	Proportion	Organism	Proportion
Urinary Tract Infections^a^	Taiwan (*N* = 3990)		South Korea (*N* = 760)		Japan (*N* = 202)	
1	*Escherichia coli*	19.8%	*Escherichia coli*	17.6%	*Escherichia coli*	37.6%
2	*Candida albicans*	16.9%	*Candida albicans*	12.6%	*Pseudomonas aeruginosa*	16.3%
3	*Enterococcus faecium*	8.5%	*Enterococcus faecalis*	9.5%	*Candida albicans*	7.9%
4	*Pseudomonas aeruginosa*	7.4%	*Enterococcus faecium*	9.3%	*Klebsiella pneumoniae*	6.9%
5	*Klebsiella pneumoniae*	7.3%	*Klebsiella pneumoniae*	8.6%	*Enterococcus faecalis*	6.4%
Bloodstream Infections	Taiwan (*N* = 4138)		South Korea (*N* = 1288)		Japan^b^ (*N* = 268)	
1	*Acinetobacter baumannii*	10.4%	*Enterococcus faecium*	14.7%	*Staphylococcus epidermidis*	15.7%
2	*Klebsiella pneumoniae*	9.6%	*Staphylococcus aureus*	14.2%	*Staphylococcus aureus*	13.0%
3	*Enterococcus faecium*	7.2%	*Acinetobacter baumannii*	12.6%	Coagulase negative staphylococci	10.1%
4	*Staphylococcus aureus*	6.5%	Coagulase negative staphylococci	12.0%	*Serratia marcescens*	5.6%
5	*Candida albicans*	6.2%	*Enterococcus faecalis*	7.3%		
Pneumonia	Taiwan (*N* = 1397)		South Korea (*N* = 554)		Japan^c^ (*N* = 650)	
1	*Pseudomonas aeruginosa*	22.5%	*Acinetobacter baumannii*	34.5%	*Staphylococcus aureus*	21.8%
2	*Acinetobacter baumannii*	18.0%	*Staphylococcus aureus*	28.5%	*Pseudomonas aeruginosa*	18.6%
3	*Klebsiella pneumoniae*	16.2%	*Klebsiella pneumoniae*	9.4%	*Klebsiella pneumoniae*	7.8%
4	*Staphylococcus aureus*	9.0%	*Pseudomonas aeruginosa*	8.8%	*Stenotrophomonas maltophilia*	6.8%
5	*Enterobacter species*	6.2%	*Enterobacter aerogenes*	3.2%		

^a^The National Healthcare Safety Network definition of catheter-associated urinary tract infections was updated in 2015, and excluded *Candida*, yeasts or molds as potential pathogens. Nevertheless, TNIS, KONIS and JANIS kept these pathogens and data are provided

^b^Japan’s data on bloodstream infection represents central line-associated bloodstream infections

^c^Japan’s data on pneumonia represents ventilator-associated pneumonia

*Escherichia coli* and *Candida albicans* were included in the top five organisms causing UTI in all three countries. *E. coli* and *C. albicans* constituted 19.8 and 16.9%, 17.6 and 12.6, 37.6 and 7.9% of UTI for Taiwan, Korea, and Japan, respectively (Table 2). Along with *Candida albicans*, non-*albicans Candida* species and yeast-like organisms constituted 31.4% of the urinary tract pathogens in Taiwan. In Korea, 23.4% of the UTI were due to *Candida* species (Additional file 3: Table S3).

*Staphylococcus aureus* was a major pathogen of BSI. The rates were 14.2% in Korea, 13.0% in Japan, and 6.5% in Taiwan (Table 2). Along with *S. aureus*, *Staphylococcus epidermidis and* coagulase-negative staphylococci constituted 38.8% of the CLABSI isolates in Japan. There were major differences among the countries in the distribution of other predominant pathogens. *Acinetobacter baumannii* and *E. faecium* were the predominant BSI pathogens in Taiwan and Korea. Major *Candida* species constituted 12.1% of the BSI isolates in Taiwan and 12.9% in Korea (Additional file 4: Table S4).

*S. aureus*, *P. aeruginosa* and *K. pneumoniae* were the predominant pathogens for HAP across all three countries (Table 2). Interestingly, *A. baumannii* was predominant in Taiwan and Korea but not in Japan, similar to the observation for BSI. For Taiwan and Korea, these four pathogens comprised 65.7 and 81.2% of the HAP isolates, respectively.

### Antimicrobial resistance

**Fig. 3:**
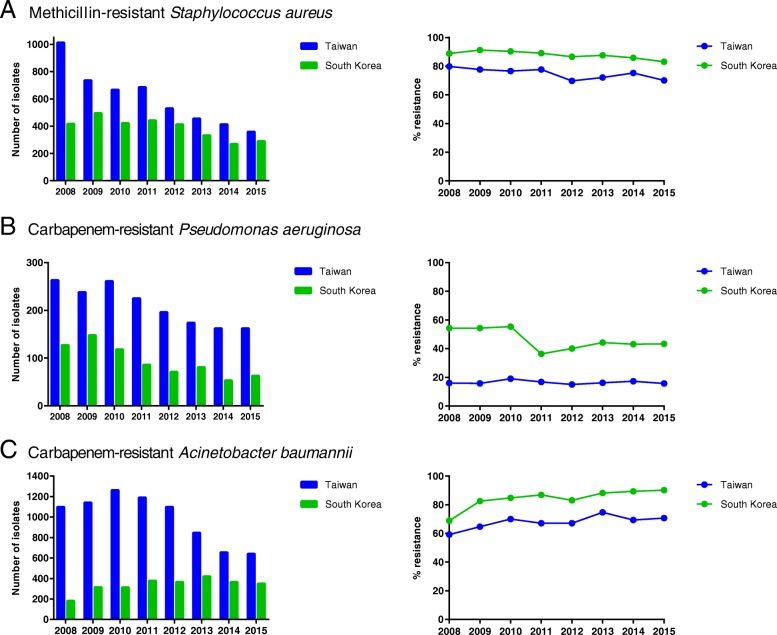
Trends of the numbers and the proportions of antimicrobial resistance in selected bacteria causing healthcare-associated infections in the intensive care units in Taiwan and South Korea. Note: The proportions of antimicrobial resistance in selected pathogens were calculated as numbers of antimicrobial-resistant isolates divided by the total numbers of isolates of the same species. Japan’s data included non-ICU patients and were not shown. **a** Methicillin-resistant *Staphylococcus aureus*; **b** Carbapenem-resistant *Pseudomonas aeruginosa*; **c** Carbapenem-resistant *Acinetobacter baumannii*

Higher AMR rates were noted in Korea than in Taiwan (Fig. 3). The data for Japan were incomplete for several study years and are not shown in the figures. There was a significant decrease in the number of isolates of methicillin-resistant *S. aureus* (MRSA) from 2008 to 2015. This included a 64.6% reduction in Taiwan and 30.5% in Korea (*p* < 0.05) (Fig. 3a). The proportion of MRSA among all *S. aureus* isolates in 2015 remained high in Korea (83.1%), while it decreased in Taiwan by 12.3% from 79.9% in 2008 to 70.1% over the 8-year period. There was also a significant decrease in the number of isolates of carbapenem-resistant *P. aeruginosa* (CRPA) during the study period with a reduction of 50.4% in Korea and 38.4% in Taiwan (*p* < 0.05) (Fig. 3b).

Carbapenem-resistant *A. baumannii* (CRAB) was more commonly isolated in Taiwan than in South Korea. The number of CRAB isolates initially increased in Taiwan and then decreased significantly with a total reduction of 49.2% from 2010 to 2015 (*p* < 0.05). In contrast, the number of CRAB isolates in Korea increased by 91.8% by the end of the study period (Fig. 3c). The proportion of CRAB among *A. baumannii* isolates was higher in Korea than in Taiwan, and increased from 2008 to 2015 in both countries.

## Discussion

In the current study, we described the surveillance and IPC programs of Taiwan, South Korea and Japan. A variation in surveillance protocol, such as HAI case definition and surveillance items was found among the three countries although these protocols were similar to those employed by the HAI-net and NHSN. There were also common IPC strategies shared by the three countries, but each with special emphasis on different aspects of IPC. We also compared the rates of HAI and the most common causative pathogens as reported by the three surveillance systems. There was a 53% decline in overall HAI in the surveyed ICUs of all three countries over the 8-year period. The overall incidence densities of HAI in Taiwan, Korea, and Japan in 2015 were 5.0, 2.8, and 2.7 per 1000 patient-days, respectively. These rates are comparable to HAI-net (2.6 per 1000 patient-days), and substantially lower than those of developing countries, as shown in Table 3 [2, 12–15].

**Table 3:** Comparison of healthcare-associated infections in intensive care units across different geographic regions

				Site-specific HAI (per 1000 patient-days)			Device-associated HAI (per 1000 device-days)		
Countries/Regions (surveillance system)	Data source or type of study	Year	Overall^a^	UTI ^b^	BSI	HAP	CAUTI^b^	CLABSI	VAP
Taiwan (TNIS)	National surveillance	2015	5.0 (8514/1692998)	2.1	2.1	0.8	3.0	3.7	1.1
South Korea (KONIS)	National surveillance	2015	2.8^c^ (2608/945605)	0.8	1.3	0.7	0.9	2.2	1.0
Japan (JANIS)	National surveillance	2015	2.7^d^ (952/347386)	0.5	–	–	–	0.7^e^	1.5^e^
USA (NHSN) [12]	National surveillance	2012	1.6^f^ (37872/23344616)	–	–	–	2.1	1.1	1.4
Europe (HAI-net) [13]	National surveillance	2015	2.6 (15821/6177114)	1.1	2.0	4.0	3.6	3.6	10.0
Southeast Asia [2]	Meta-analysis^g^	2000–2012	20.0^h^ (16.9450/26681)	–	–	–	8.9	4.7	14.7
Developing countries worldwide [14]	Meta-analysis^g^	1995–2008	47.9^h^ (28.54250/148893)	–	–	–	9.8	11.3	22.9
Developing countries worldwide (INICC)^i^ [15]	Multi-center study	2010–2015	–	–	–	–	5.1	4.1	13.1

*Abbreviations: BSI* bloodstream infections, *CAUTI* catheter-associated urinary tract infections, *CLABSI* central line-associated bloodstream infections, *HAI* healthcare-associated infections, *HAI-net* Healthcare-associated Infections Surveillance Network (Europe), *HAP* hospital-acquired pneumonia, *ICU* intensive care units, *INICC* International Nosocomial Infection Control Consortium (developing countries worldwide), *NHSN* National Healthcare Safety network (USA), *UTI* urinary tract infections, *VAP* ventilator-associated pneumonia

^a^Data are pooled mean of site-specific HAI such as UTI, BSI, and HAP or otherwise specified, and computed from raw data provided in the reports. Thus, all these data should be interpreted appropriately

^b^The NHSN CAUTI definition was updated in 2015 and excluded *Candida*, yeasts or molds as potential CAUTI pathogens. Nevertheless, TNIS, KONIS and JANIS kept these pathogens and data are provided

^c^Data were collected during July 2015–June 2016

^d^Data are pooled means of UTI, CLABSI and VAP

^e^Data were calculated by episodes/1000 patient-day

^f^Data are pooled means of CAUTI, CLABSI and VAP

^g^Infection frequencies reported in high-quality studies were greater than those from low-quality studies

^h^Weights were given to different studies to compute the final data. Unweighted raw data were derived from the original article and denoted in parenthesis

^i^Data were prospectively collected from 861,284 patients in 703 ICUs from 50 countries

We believe that essential elements that contributed to the sustained decrease in the incidence of HAI in Taiwan, Korea and Japan were the national surveillance programs combined with improvement in IPC practices [16]. In Korea, there was a significant decline in device-associated HAI in association with the implementation of the KONIS program [17]. National IPC programs such as hand hygiene, care bundles, antimicrobial stewardships, and environmental hygiene have been shown to effectively reduce HAI and infections caused by AMR pathogens [7, 18–20]. In Taiwan, hand hygiene program over a 4-year period were found to reduce HAI in ICU by 17.2% and BSI by 12.7% [20], and care bundles to further reduce CAUTI and CLABSI by 22.7 and 12.2%, respectively [21, 22]. In Korea, a multicentre study found that VAP rate decreased from 4.08 to 1.16 cases per 1000 ventilator-days following 3 months of bundle intervention [23]. Evidence-based IPC practices have been shown to be cost-saving and effective in preventing HAI [20, 24]. Adoption of these practices to reduce HAI burden might be helpful for many Asian countries, which are facing problems such as rising healthcare costs and inefficient healthcare insurance systems [25].

Appointment of infection control professionals or infection control committees is a common strategy across the three countries (Fig. 1, Additional file 2: Table S2). In Japan, one serious fundamental obstacle before 2010 was the lack of personnel dedicated to IPC. In 2010, Japan revised medical reimbursement system and provided 10 USD per patient per admission if a hospital payed the annual cost for the designated work hours for infection control personnel which included certificated nurse, doctor, pharmacist and medical technician/microbiologist. IPC incentives through reimbursement policies were revised in 2012 and 2018, as described in Additional file 2: Table S2. Such a scheme encourages hospitals dedicated certificated personnel to participate in IPC (Table 1). Other than manpower, personnel training and resource infrastructure are essential for surveillance and prevention of HAI [26, 27]. During the study period, financial incentives to support IPC programs were employed by Korea and Japan. Japan switched its reimbursement system to a penalty system in 2000, and then changed it back to the current reward system in 2010. This suggests that a supportive environment that encourages IPC practices might be better than one that punishes for wrongdoing, and should be fostered by national authorities for effective prevention of HAI [27]. Correspondingly, such a difference in reimbursement may have well influenced the outcomes of HAI in Taiwan, Korea and Japan.

Changes in case definition might have contributed to the observed HAI trends. For example, the newer definition for UTI established in 2009 would probably have excluded cases that might have been classified as HAI under the older definition [28]. Nevertheless, based on the consistent decline of HAI incidence across all infection categories, it is unlikely that modifications in case definition can explain the remarkable decrease in HAI trends.

Substantial variation exists for causative pathogens of HAI across the three countries. This variability could be due to a number of factors, including baseline characteristics of participating hospitals and ICUs, variation in diagnostic standards and case definitions, geography and climate, and IPC practices. For example, Japan’s BSI was dominated by staphylococci (39.9%) possibly because its reports were limited to device-associated modules in BSI. An interesting variation that is likely not attributable to systemic differences was noted for *A. baumannii*, which was isolated commonly from Taiwan and Korea but rarely from Japan. Results from HAI-net seem to support this notion, with higher proportions of HAI caused by *Acinetobacter spp.* in some countries [13].

Our study showed a general decrease in isolates of important AMR species: MRSA, CRPA and CRAB even though the number of participating ICUs has expanded from 2008 to 2015. This downward trend is likely due to hand hygiene to prevent cross-transmission of AMR pathogens, care bundles to prevent device- or procedure-associated infections, and antimicrobial stewardship programs to mitigate the selection pressure implemented in these countries [7, 18–20, 29]. A recent meta-analysis reported that antimicrobial stewardship programs in Asia reduced overall antimicrobial consumption by 9.74% and incidence density of important AMR pathogens such as MRSA by 0.9 to 1.4 isolates per 1000 patient-days [19]. Expenditure associated with antimicrobial prescription and hospitalization were also found to decrease by a range of 9.7 to 58.1%. These findings highlight the efficacy and importance of antimicrobial stewardship programs in combating the rise of AMR pathogens.

While surveillance of HAI may provide important feedback for IPC efforts, the high costs in establishing and maintaining the system may preclude many countries from undertaking such an ordeal. Introducing information technology in surveillance systems may help reduce labor intensive and increase the efficiency of surveillance [30–33]. In Asia, National Taiwan University Hospital has established a web-based real-time surveillance system based on algorithms for AMR pathogens, UTI and BSI. The surveillance system is sophisticated in its ability to integrate and analyse several data sources [32, 33]. Their studies and a recent systematic review of the literature demonstrated that adopting electronic surveillance software yields considerable time savings pertaining to case findings, data collection, case ascertainment and classification while maintaining high levels of sensitivity and specificity [31–33]. Thus, information technology may represent an opportunity for countries seeking to establish HAI surveillance and overcome the gaps of human resources.

Our study provides a framework for other countries to establish or improve surveillance and IPC programs. Further studies on cost-effectiveness of these strategies will be helpful to relevant stakeholders as they allocate and prioritize budget for infection control. Our work also serves as the foundation for possible regional collaborations in East Asia or in greater Asia. Standardization of protocols will allow inter-country comparison and benchmarking. For Europe, the similarities and differences in HAI trends between our study and the HAI-net re-affirmed the need for continual surveillance and IPC efforts.

The strengths of this study were our ability to obtain an overview of the surveillance and IPC programs of Taiwan, Korea, and Japan that were seldom described in past reports. We were also able to obtain comprehensive HAI data from their surveillance systems and compare these data with Western developed countries and developing countries worldwide. The limitations were the need to use open access datasets. This restricted our ability to assess and compare HAI epidemiology comprehensively across the three countries. JANIS only releases data on UTI, CLABSI, and VAP in its surveillance reports. Information on the other infection modules: CAUTI, BSI, and HAP were unavailable. We were unable to comprehensively describe the complete pathogen rankings and AMR profiles for each country, because these data were unavailable on some surveillance systems. There are differences in protocols employed by each surveillance system, such as JANIS, which calculated device-associated infections differently. Standardization of protocols should allow for inter-country comparison. Furthermore, we need to include antibiotic use in future studies because of their critical impact on development of resistance. Finally, there were differences in the types of the hospitals enrolled in the three systems, wide variation of hospital participation rates (6.8% in Japan and 100% in Taiwan), and thus, discrepancies in hospital coverage rates (1.9% in Japan and 21.2% in Taiwan). Therefore, data presented here cannot be generalized to the entire 3 countries.

## Conclusions

We found that national surveillance data obtained from Taiwan, South Korea, and Japan from 2008 to 2015 was associated with a 53.0% reduction in HAI in surveyed ICUs. There were differences among the countries in surveillance protocols, hospital coverage rates, national IPC programs, distribution of invading microorganisms and antibiotic resistance. The overall decrease in HAI appears to be due to improved surveillance coupled with a series of interventions in each country. We propose that a regional HAI network be established in East Asia similar to Europe’s HAI-net. Such a coordinated effort should enable greater regional collaborations and development of joint strategies as we learn from one another.

## Additional files

Additional file 1: **Table S1.** The number of participating hospitals and intensive care units of national healthcare-associated infection surveillance system in each country from 2008 to 2015. (DOCX 33 kb)

Additional file 2: **Table S2.** National infection prevention and control policies and programs initiated, implemented or extended in Taiwan, South Korea, and Japan. (DOCX 59 kb)

Additional file 3: **Table S3.** Common causative pathogens of healthcare-associated urinary tract infections in intensive care units enrolled in national surveillance systems in Taiwan, South Korea, and Japan in 2015. (DOCX 36 kb)

Additional file 4: **Table S4.** Common causative pathogens of healthcare-associated bloodstream infections in intensive care units enrolled in national surveillance systems in Taiwan, South Korea, and Japan in 2015. (DOCX 36 kb)

Additional file 5: **Table S5.** Common causative pathogens of healthcare-associated pneumonia in intensive care units enrolled in national surveillance systems in Taiwan, South Korea, and Japan in 2015. (DOCX 36 kb)

## Abbreviations

AMR: Antimicrobial resistance
AS: Antimicrobial stewardship
BSI: Bloodstream infection
CAUTI: Catheter-associated urinary tract infection
CLABSI: Central line-associated bloodstream infection
CRPA: Carbapenem-resistant *Pseudomonas aeruginosa* CRAB Carbapenem-resistant *Acinetobacter baumannii*
FTE: Full-time equivalent
HAI: Healthcare-associated infections
HAI-net: European Healthcare-associated Infections Surveillance Network
HAP: Hospital-acquired pneumonia
IC: Infection control
ICD: Infection control doctor
ICN: Infection control nurse
ICP: Infection control personnel
ICU: Intensive care units
INICC: International Nosocomial Infection Control Consortium
IPC: Infection prevention and control
JANIS: Japan Nosocomial Infection Surveillance
KONIS: Korean National Healthcare-associated Infection Surveillance
MRSA: Methicillin-resistant *Staphylococcus aureus*
NHSN: National Healthcare Safety Network
TNIS: Taiwan Nosocomial Infection Surveillance
UTI: Urinary tract infection
VAP: Ventilator-associated pneumonia
