# Supplementary material for: Healthcare-associated infections in intensive care units in Taiwan, South Korea, and Japan: recent trends based on national surveillance reports
Source: Antimicrob Resist Infect Control. 2018 Nov 7;7:129. doi: 10.1186/s13756-018-0422-1 (PMC6223041; doi:10.1186/s13756-018-0422-1)
Supplement: Supplementary file 1 — Table S1. The number of participating hospitals and intensive care units of national healthcare-associated infection surveillance system in each country from 2008 to 2015. (DOCX 33 kb) [file 13756_2018_422_MOESM1_ESM.docx]

**Table S1. The number of participating hospitals and intensive care units of national healthcare-associated infection surveillance system in each country from 2008 to 2015.**

| **A. Number of hospitals** | | | | | | | | |
| --- | --- | --- | --- | --- | --- | --- | --- | --- |
| **Country** | **2008** | **2009** | **2010** | **2011** | **2012** | **2013** | **2014** | **2015** |
| **Taiwan** | 65 | 83 | 104 | 105 | 103 | 102 | 103 | 104 |
| **South Korea** | 57 | 63 | 72 | 81 | 91 | 94 | 96 | 103 |
| **Japan** | 103 | 104 | 95 | 106 | 106 | 124 | 143 | 164 |
|  | |  |  |  |  |  |  |  |
| **B. Number of intensive care units** | | | | | | | | |
| **Country** | **2008** | **2009** | **2010** | **2011** | **2012** | **2013** | **2014** | **2015** |
| **Taiwan** | 453 | 476 | 498 | 465 | 460 | 459 | 472 | 475 |
| **South Korea** | 101 | 116 | 130 | 143 | 161 | 166 | 169 | 178 |
| **Japan** | 184 | 203 | 153 | 161 | 158 | 152 | 163 | 187 |
