# Supplementary material for: Healthcare-associated infections in intensive care units in Taiwan, South Korea, and Japan: recent trends based on national surveillance reports
Source: Antimicrob Resist Infect Control. 2018 Nov 7;7:129. doi: 10.1186/s13756-018-0422-1 (PMC6223041; doi:10.1186/s13756-018-0422-1)
Supplement: Supplementary file 2 — Table S2. National infection prevention and control policies and programs initiated, implemented or extended in Taiwan, South Korea, and Japan. (DOCX 59 kb) [file 13756_2018_422_MOESM2_ESM.docx]

**Table S2.** National infection prevention and control policies and programs initiated, implemented or extended in Taiwan, South Korea, and Japan.

| **Country** | **Intervention** | **Description** |
| --- | --- | --- |
| **Taiwan** | Policy for national infection prevention and control program | Since 1984, the Ministry of Health (now Ministry of Health and Welfare) initiated the national IPC program, which included healthcare staff training, infection control committee and hospital policies [1]. Since 2004, national actions to enhance IPC activities included organizational changes and incentives provided by the national health insurance program, enhanced IPC and strengthening the preparedness of emerging infectious diseases in frontline healthcare facilities [2]. |
|  | Infectious Disease Prevention Act on ICP staffing | Since 1984, every teaching hospital is required to have one full-time equivalent ICN per 300 hospital beds [1]. Since 2004, hospitals with more than 500 beds are required to have at least one ICD, and hospitals with more than 300 beds are required to have at least one ICN per 250 beds [2]. In 2017, hospitals with more than 500 beds are encouraged to have one ICD for every 300 beds and one ICN for every 250 beds [3]. |
|  | Audit and accreditation | Since 1984, the Ministry of Education has included IPC as an element of teaching hospital accreditation. Since 1988, the medical service act has mandated the assessment of IPC practices in hospital accreditation [1]. Annual nationwide hospital audit for IPC quality was extended stepwise to cover all hospitals since 2005. For objective evaluation of the quality of IPC in each hospital, a comprehensive checklist has been developed and are revised periodically. The checklist takes into consideration of the type and size of the hospital. The results are integrated into performance evaluation during the hospital accreditation process. Items of the checklist include infrastructure such as infection control committee and ICP, policies and interim guidelines, and quality improvement programs. |
|  | Taiwan nosocomial infection surveillance | Paperless surveillance system of HAI involved medical centers since October 2001 and regional hospitals since July 2002. Current web-based system was launched in 2007. AMR detection and reporting system began in 2007 [4]. TNIS system adopts voluntary reporting, and each hospital may provide their data either through web-based entry or convey their data electronically through interchange platform. |
|  | National campaign on Hand hygiene | This is the first of a series of nationwide interventional programs promoted by the Taiwan CDC. The pilot study was initiated in 2007 in three Centers of Excellence on hand hygiene following a successful hospital-wide hand hygiene program at National Taiwan University Hospital [5]. WHO multimodal strategy was adapted and program was expanded to the national level in 2010. Theme of WHO’s “Clean Care is Safer Care” is promoted via the program. Culture of patient safety formed via nationwide hand hygiene certification. Included in hospital accreditation for continuous quality improvement [6]. |
|  | National campaigns on care bundles | The pilot study for CLABSI care bundle was initiated in 2011 and expanded to nationwide in 2013. The CAUTI and VAP bundles was initiated in 2013 and expanded to national level in 2015. |
|  | Hospital environmental hygiene program | Pilot study began in 2012 and was expanded to national policy in 2015. The program focused on increasing hospital cleanliness and reducing antimicrobial-resistant organisms. |
|  | Antimicrobial stewardship program | The Bureau of National Health Insurance issued a new reimbursement regulation effective from 1 February 2001 forbidding the use of antimicrobials in ambulatory patients with upper respiratory infections but without evidence of bacterial infection [7]. A recent multifaceted national antimicrobial stewardship program (2013-2015) included a national task force, AMR management strategies, surveillance of HAI, selected AMR bacteria and the antimicrobial use, continuation of aforementioned hospital IPC programs, and regulation of pharmaceutical affairs [8, 9]. |
| **South Korea** | Antimicrobial stewardship program | In order to encourage appropriate prescription of antibiotics in medical institutions, the government has implemented a series of healthcare policies, consisting of legislative separation of drug prescribing and dispensing, antibiotic utilization reviews, healthcare quality assessment, and public reporting since 2000 [10]. |
|  | National guidelines on infection control | Guidelines for healthcare-associated infection prevention and control program were developed by the government and implemented in 2002 [11]. |
|  | National healthcare-associated infection surveillance | KONIS began in 2006 [12]. A recent study showed that KONIS data are generally reliable; however, KONIS validation studies found a decrease in sensitivity for BSIs in 2014 compared to 2008 and 2010 [13]. |
|  | Antimicrobial resistance management | The National AMR Safety Management Program has been implemented since 2003. Surveillance of AMR organisms was included in 2010 [14]. A new National AMR Management Action Plan (2016-2020) has been established in response to the WHO’s Global Action on AMR [11, 15]. |
|  | Hand hygiene program | Pilot study initiated in 35 hospitals (out of 90 hospitals participating in KONIS) in 2013 [16]. |
|  | Medical Service Act to mandate infection control and surveillance | Acts 29 and 47 were passed in 2002. Act 29 specifies that hospital infection surveillance and controls were the duties of hospitals with more than 300 beds. Act 47 specifies that hospital infection surveillance and controls are legislated for the accreditation of quality of care every three years starting from 2004 [17]. In 2012, hospitals with more than 200 beds are required to appoint an infection control committee and at least one full-time experienced staff to oversee the IPC program. |
|  | Infection control subsidy program | In 2016, as financial incentives for healthcare facilities that actively participate in infection surveillance systems and IPC activities, a reimbursement of 1,950-2,870 Won (1.8-2.7 USD) per patient per admission was introduced. |
| **Japan** | Japan nosocomial infection surveillance | JANIS was established in 2000 and reformed in 2008 as a voluntary-based national surveillance system targeting HAI and AMR bacteria [18]. According to a national survey involving 971 hospitals in 2012, 67.3%, 35.1% and 31.5% of 685 respondents regularly monitor rates of CLABSI, CAUTI and VAP, respectively [19]. JANIS collects data from hospital laboratories that have an automated system for bacterial identification and drug susceptibility testing, and also data from commercial laboratories to which participating hospitals are contracted. |
|  | Penalties and reimbursement policies | In 1996, hospitals with good IPC practices were reimbursed 0.6 USD per patient per day. In 2000, reimbursement policy was replaced with penalties for hospitals with insufficient IPC policies. This policy was discontinued in 2006.  Medical reimbursement system was revised in 2010 [18] and upgraded in 2012. This revision aimed to provide IPC incentive and mainly focused on manpower. Since 2012, each hospital is reimbursed 1000 JPY (about 10 USD) per patient per admission if it fulfills the Ministry of Health, Labor and Welfare requirements, which mandated one dedicated full-time certificated ICN (at >0.8 FTE), one part-time ICD (at >0.5 FTE), one part-time IC pharmacist and one part-time medical technician/microbiologist (at >0.5 FTE).  IPC incentive through reimbursement policies have been upgraded again in 2018. Since 2018, reimbursement policies per admission included three parts. It provides 3900 JPY (about 39 USD) per admission for IPC incentive at a major hospital, or 1000 JPY for a small hospital. Additional 1000 JPY was reimbursed if this hospital participates in a local IPC network incentive. Another 1000 JPY was reimbursed for AS incentive. For hospitals with AS incentive, it mandates the following manpower in addition to 2012 requirements: one part-time doctor mainly for AS (at >0.5 FTE), one full-time ICP either a certificated ICN or IC pharmacist or medical technician. |
|  | Medical Service Act | Mandatory assignment of dedicated ICP at advanced treatment hospitals in 2004 [18].  In 2007, Japanese medical law obligated all health care institutions to implement operational safety measures against HAIs. Core components include hospital IPC guidelines, implementing employee IPC training, and disease reporting [18]. In 2011, the Ministry of Health, Labor and Welfare issued an official ordinance containing guidance on establishing IPC teams within health care facilities, criteria for disease reporting, and collaboration between institutions for complex cases. The ordinance’s 2012 medical fee revision raised the subject of hospital fees for IPC efforts. |
|  | Infection prevention and control practices implementation | A national survey in 2012 showed a wide variation in adoption of IPC practices for CLABSI, CAUTI and VAP among 685 respondent hospitals [19]. |
|  |  |  |

**Abbreviations:** AMR: antimicrobial-resistance; AS: antimicrobial stewardship; BSI: bloodstream infections; CAUTI: catheter-associated urinary tract infections; CDC: Centers for Disease Control; CLABSI: central line-associated bloodstream infections; HAI: healthcare-associated infections; FTE: full-time equivalent; HAP: hospital-acquired pneumonia; IC: infection control; ICD: infection control doctor; ICN: infection control nurse; ICP: Infection control personnel; ICU: intensive care units; IPC: Infection prevention and control; JANIS: Japan nosocomial infection surveillance; KONIS: Korean national healthcare-associated infection surveillance; MDRO: multi-drug resistant organisms; TNIS: Taiwan nosocomial infection surveillance; UTI: urinary tract infections; VAP: ventilator-associated pneumonia.

**References:**

1. Centers for Disease Control, R.O.C (Taiwan). Infection Control Manual for Medical Care Facilities. 2014. Available at: <https://www.cdc.gov.tw/professional/list.aspx?treeid=beac9c103df952c4&nowtreeid=52e2faab2576d7b1>. Accessed 24 March 2018.

2. Centers for Disease Control, R.O.C (Taiwan). Regulation of infection control in medical facilities. 2008. Available at: [www.cdc.gov.tw/professional/downloadfile.aspx?fid=9F265771F1EE004E](http://www.cdc.gov.tw/professional/downloadfile.aspx?fid=9F265771F1EE004E). Accessed 24 March 2018.

3. Centers for Disease Control, R.O.C (Taiwan). Regulation of infection control in medical facilities. 2017. Available at: <https://www.cdc.gov.tw/professional/info.aspx?treeid=beac9c103df952c4&nowtreeid=bd387fa55fef03f0&tid=DBE92A531EC6E44A>. Accessed 24 March 2018.

4. UPMC Center for Health Security. IHR Joint External Evaluation of Taiwan. 2016. Available at: <http://www.cdc.gov.tw/uploads/files/201701/cbf247f3-64b0-4675-a5e8-d4f70eaf24e5.pdf>. Accessed 24 March 2018.

5. Chen YC, Sheng WH, Wang JT et al. Effectiveness and Limitations of Hand Hygiene Promotion on Decreasing Healthcare–Associated Infections. PLoS One, 2011; 6:e27163.

6. Centers for Disease Control, R.O.C (Taiwan). Hand Hygiene. Available at: <https://www.cdc.gov.tw/professional/HandHygiene.aspx?theme=HandHygiene&treeid=15ea1948ffc4fa7a&nowtreeid=46C503C6997FECF3>. Accessed 24 March 2018.

7. Ho M, Hsiung CA, Yu HT, Chi CL, Chang HJ. Changes before and after a policy to restrict antimicrobial usage in upper respiratory infections in Taiwan. Int J Antimicrob Agents, 2004; 23:438-445.

8. Tseng SH, Lee CM, Lin TY et al. Combating antimicrobial resistance: Antimicrobial stewardship program in Taiwan. J Microbiol Immunol Infect, 2012; 45:79-89.

9. Centers for Disease Control, R.O.C (Taiwan). National Action Plan for Antimicrobial Stewardship Program. 2015. Available at: <https://www.cdc.gov.tw/uploads/files/201601/fa3be5b4-7a0d-4ed1-b930-1eb97f711b7c.pdf>. Accessed 24 March 2018.

10. Kim BN, Kim HB, Oh MD. Antibiotic control policies in South Korea, 2000-2013. Infect Chemother, 2016; 48:151-159.

11. World Health Organization. Joint external evaluation of IHR core capacities of the Republic of Korea. 2017. Available at: <https://extranet.who.int/spp/sites/default/files/jeeta/WHO-WHE-CPI-2017.65-eng.pdf>. Accessed 24 March 2018.

12. Oh HS, Cheong HW, Yi SE, Kim H, Choe KW, Cho SI. Development and Application of Evaluation Indices for Hospital Infection Surveillance and Control Programs in the Republic of Korea. Infect Control Hosp Epidemiol, 2007; 28:435-445.

13. Kwak YG, Choi JY, Yoo HM, et al. Validation of the Korean National Healthcare-associated Infections Surveillance System (KONIS): an intensive care unit module report. J Hosp Infect, 2017; 96:377-384.

14. Yoon YK, Lee SE, Seo BS et al. Current status of personnel and infrastructure resources for infection prevention and control programs in the Republic of Korea: A national survey. Am J Infect Control, 2016; 44:e189-e193.

15. Ryu S. The new Korean action plan for containment of antimicrobial resistance. J Glob Antimicrob Resist, 2017; 8:70-73.

16. Kim HB. National hand hygiene campaign in Korea-progress and challenges. J Microbiol Immunol Infect, 2015; 48:S11.

17. Oh HS, Chung HW, Kim JS, Cho SI. National survey of the status of infection surveillance and control programs in acute care hospitals with more than 300 beds in the Republic of Korea. Am J Infect Control, 2006; 34:223-233. .

18. Morikane K. Infection control in healthcare settings in Japan. J Epidemiol, 2012; 22:86-90.

19. Krein SL, Greene MT, Apisarnthanarak A et al. Infection prevention practices in Japan, Thailand, and the United States: results from national surveys. Clin Infect Dis, 2017; 64(suppl_2):S105-S111.
