# Supplementary material for: Healthcare-associated infections in intensive care units in Taiwan, South Korea, and Japan: recent trends based on national surveillance reports
Source: Antimicrob Resist Infect Control. 2018 Nov 7;7:129. doi: 10.1186/s13756-018-0422-1 (PMC6223041; doi:10.1186/s13756-018-0422-1)
Supplement: Supplementary file 3 — Table S3. Common causative pathogens of healthcare-associated urinary tract infections in intensive care units enrolled in national surveillance systems in Taiwan, South Korea, and Japan in 2015. (DOCX 36 kb) [file 13756_2018_422_MOESM3_ESM.docx]

**Table S3. Common causative pathogens of healthcare-associated urinary tract infections in intensive care units enrolled in national surveillance systems in Taiwan, South Korea, and Japan in 2015.**

|  | Taiwan (N= 3990) | | South Korea (N=760) | | Japan (N=202) | |
| --- | --- | --- | --- | --- | --- | --- |
| Rank | **Organism** | **Proportion** | **Organism** | **Proportion** | **Organism** | **Proportion** |
| 1 | *Escherichia coli* | 19.8% | *Escherichia coli* | 17.6% | *Escherichia coli* | 37.6% |
| 2 | *Candida albicans* | 16.9% | *Candida albicans* | 12.6% | *Pseudomonas aeruginosa* | 16.3% |
| 3 | *Enterococcus faecium* | 8.5% | *Enterococcus faecalis* | 9.5% | *Candida albicans* | 7.9% |
| 4 | *Pseudomonas aeruginosa* | 7.4% | *Enterococcus faecium* | 9.3% | *Klebsiella pneumoniae* | 6.9% |
| 5 | *Klebsiella pneumoniae* | 7.3% | *Klebsiella pneumoniae* | 8.6% | *Enterococcus faecalis* | 6.4% |
| 6 | Yeast-like organisms | 7.3% | *Candida tropicalis* | 7.6% |  |  |
| 7 | Non-*albicans Candida* species | 7.2% | *Pseudomonas aeruginosa* | 7.2% |  |  |
| 8 | *Acinetobacter baumannii* | 4.2% | *Acinetobacter baumannii* | 4.5% |  |  |
| 9 | *Enterobacter* species | 2.4% | *Candida glabrata* | 3.2% |  |  |
| 10 | *Staphylococcus aureus* | 1.1% | Coagulase negative staphylococci | 2.9% |  |  |

Note. Data were limited to top 5 causative pathogens in Japan. Yeast-like organisms were not further identified.
