# Supplementary material for: Healthcare-associated infections in intensive care units in Taiwan, South Korea, and Japan: recent trends based on national surveillance reports
Source: Antimicrob Resist Infect Control. 2018 Nov 7;7:129. doi: 10.1186/s13756-018-0422-1 (PMC6223041; doi:10.1186/s13756-018-0422-1)
Supplement: Supplementary file 4 — Table S4. Common causative pathogens of healthcare-associated bloodstream infections in intensive care units enrolled in national surveillance systems in Taiwan, South Korea, and Japan in 2015. (DOCX 36 kb) [file 13756_2018_422_MOESM4_ESM.docx]

**Table S4. Common causative pathogens of healthcare-associated bloodstream infections in intensive care units enrolled in national surveillance systems in Taiwan, South Korea, and Japan in 2015.**

|  | Taiwan (N=4138) | | South Korea (N=1288) | | Japan (N=268) | |
| --- | --- | --- | --- | --- | --- | --- |
| Rank | **Organism** | **Proportion** | **Organism** | **Proportion** | **Organism** | **Proportion** |
| 1 | *Acinetobacter baumannii* | 10.4% | *Enterococcus faecium* | 14.7% | *Staphylococcus epidermidis* | 15.7% |
| 2 | *Klebsiella pneumoniae* | 9.6% | *Staphylococcus aureus* | 14.2% | *Staphylococcus aureus* | 13.0% |
| 3 | *Enterococcus faecium* | 7.2% | *Acinetobacter baumannii* | 12.6% | Coagulase negative staphylococci | 10.1% |
| 4 | *Staphylococcus aureus* | 6.5% | Coagulase negative staphylococci | 12.0% | *Serratia marcescens* | 5.6% |
| 5 | *Candida albicans* | 6.2% | *Enterococcus faecalis* | 7.3% |  |  |
| 6 | Non-*albicans Candida* species | 5.9% | *Candida albicans* | 6.5% |  |  |
| 7 | *Enterobacter* species | 5.4% | *Klebsiella pneumoniae* | 4.7% |  |  |
| 8 | *Pseudomonas aeruginosa* | 5.2% | *Candida tropicalis* | 3.5% |  |  |
| 9 | *Escherichia coli* | 4.5% | *Pseudomonas aeruginosa* | 3.2% |  |  |
| 10 | Coagulase negative staphylococci | 2.5% | *Candida glabrata* | 2.9% |  |  |

Note. Data comprised only central line-associated bloodstream infections and were limited to top 5 causative pathogens in Japan.
