# Supplementary material for: Healthcare-associated infections in intensive care units in Taiwan, South Korea, and Japan: recent trends based on national surveillance reports
Source: Antimicrob Resist Infect Control. 2018 Nov 7;7:129. doi: 10.1186/s13756-018-0422-1 (PMC6223041; doi:10.1186/s13756-018-0422-1)
Supplement: Supplementary file 5 — Table S5. Common causative pathogens of healthcare-associated pneumonia in intensive care units enrolled in national surveillance systems in Taiwan, South Korea, and Japan in 2015. (DOCX 36 kb) [file 13756_2018_422_MOESM5_ESM.docx]

**Table S5. Common causative pathogens of healthcare-associated pneumonia in intensive care units enrolled in national surveillance systems in Taiwan, South Korea, and Japan in 2015.**

|  | Taiwan (N=1397) | | South Korea (N=554) | | Japan (N=650) | |
| --- | --- | --- | --- | --- | --- | --- |
| Rank | **Organism** | **Proportion** | **Organism** | **Proportion** | **Organism** | **Proportion** |
| 1 | *Pseudomonas aeruginosa* | 22.5% | *Acinetobacter baumannii* | 34.5% | *Staphylococcus aureus* | 21.8% |
| 2 | *Acinetobacter baumannii* | 18.0% | *Staphylococcus aureus* | 28.5% | *Pseudomonas aeruginosa* | 18.6% |
| 3 | *Klebsiella pneumoniae* | 16.2% | *Klebsiella pneumoniae* | 9.4% | *Klebsiella pneumoniae* | 7.8% |
| 4 | *Staphylococcus aureus* | 9.0% | *Pseudomonas aeruginosa* | 8.8% | *Stenotrophomonas maltophilia* | 6.8% |
| 5 | *Enterobacter species* | 6.2% | *Enterobacter aerogenes* | 3.2% |  |  |
| 6 | *Escherichia coli* | 4.7% | *Streptococcus species* | 2.5% |  |  |
| 7 | *Candida albicans* | 2.4% | *Escherichia coli* | 2.2% |  |  |
| 8 | Yeast-like organisms | 0.6% | *Enterobacter cloacae* | 2.2% |  |  |
| 9 | Non-*albicans Candida* species | 0.4% | *Candida albicans* | 1.8% |  |  |
| 10 | Coagulase negative staphylococci | 0.2% | *Stenotrophomonas maltophilia* | 1.1% |  |  |

Note. Data comprised only ventilator-associated pneumonia and were limited to top 5 causative pathogens in Japan
